# Supplementary material for: Axonal TDP-43 condensates drive neuromuscular junction disruption through inhibition of local synthesis of nuclear encoded mitochondrial proteins
Source: Nat Commun. 2021 Nov 25;12:6914. doi: 10.1038/s41467-021-27221-8 (PMC8617040; doi:10.1038/s41467-021-27221-8)
Supplement: Supplementary file 1 — Supplementary Information [file 41467_2021_27221_MOESM1_ESM.pdf]

- Supplementary Information -

Axonal TDP-43 condensates drive neuromuscular junction disruption through inhibition of local synthesis of nuclear encoded mitochondrial proteins

Topaz Altman[1]#, Ariel Ionescu[1]#, Amjad Ibraheem[1], Dominik Priesmann[2], Tal Gradus-Pery[1], Luba Farberov[1], Gayster Alexandra[3], Natalia Shelestovich[3], Ruxandra Dafinca[4], Noam Shomron[1,5], Florence Rage[6], Kevin Talbot[4], Michael E Ward[7], Amir Dori[8], Marcus Krüger[2] and Eran Perlson[1,5]*

[1] Sackler Faculty of Medicine, Tel-Aviv University, Tel-Aviv, Israel.

[2] CECAD Research Center and Center for Molecular Medicine (CMMC), University of Cologne, 50931, Cologne, Germany

[3] Pathology Institute, Sheba Medical Center, Tel Hashomer, Ramat Gan, Israel

[4] Nuffield Department of Clinical Neurosciences, University of Oxford, Oxford, UK.

[5] Sagol School of Neuroscience, Tel-Aviv University, Tel-Aviv, Israel.

[6] Institut de Génétique Moléculaire de Montpellier,IGMM UMR535.

[7] National Institute of Neurological Disorders and Stroke, National Institutes of Health, Bethesda, MD, USA.

[8] Department of Neurology, Sheba Medical Center, Tel Hashomer and Sackler Faculty of Medicine, Tel Aviv University, Ramat Gan, Israel

# These authors contributed equally to this work

*Corresponding author:

Eran Perlson, Ph.D., Dept. of Physiology and Pharmacology, Sackler Faculty of Medicine, Room 605, Sagol School of Neuroscience, Tel Aviv University, Ramat Aviv, Tel Aviv 69978, Israel. +972-3-6408743

E-mail: eranpe@tauex.tau.ac.il

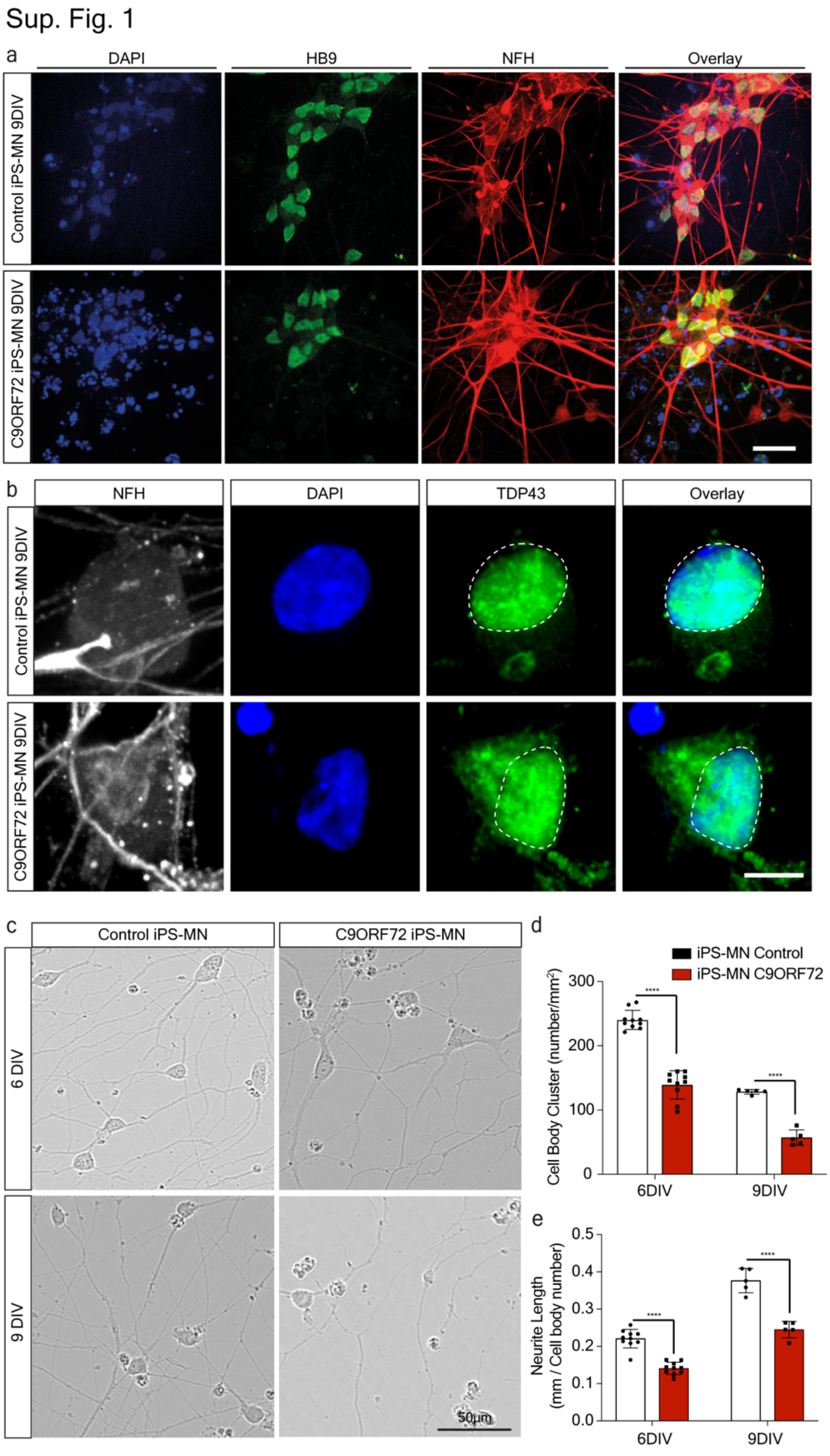

Supplementary figure 1 – Differentiation and characterization on C9ORF72 ALS patient-derived iPS-MN and corrected isogenic control.

a) Representative immunofluorescent images of C9ORF72-mutated and isogenic control iPS-MN at 9-days in vitro (DIV) demonstrating Doxycylin-induced differentiation into MN. DAPI (Blue). HB9 (Green). NFH (Red). Scale bar=50µm. n=3 independent experiments. **b)** Representative immunofluorescent images of TDP43 cytoplasmic mislocalization in C9ORF72 iPS-MN. Grey indicates NFH, blue indicates nuclei (DAPI), green indicates TDP-43. Scale bar=10µm. n=3 independent experiments. **c)**

Representative bright-field images and quantification **(d-e)** demonstrating differences in neurite length between C9ORF72 iPS-MN and their isogenic controls. Quantification of the **d)** number of cell-body cluster per mm^2 and **e)** the neurite length in mm per cell body cluster in C9ORF72 iPS-MN and isogenic control. Scale bar=50μm. n=10 wells for 6-DIV, and n=5 for 9-DIV wells. SD. Unpaired t-test, two-sided. ****p<0.0001. Source data are provided as a Source Data file.

Sup. Fig. 2

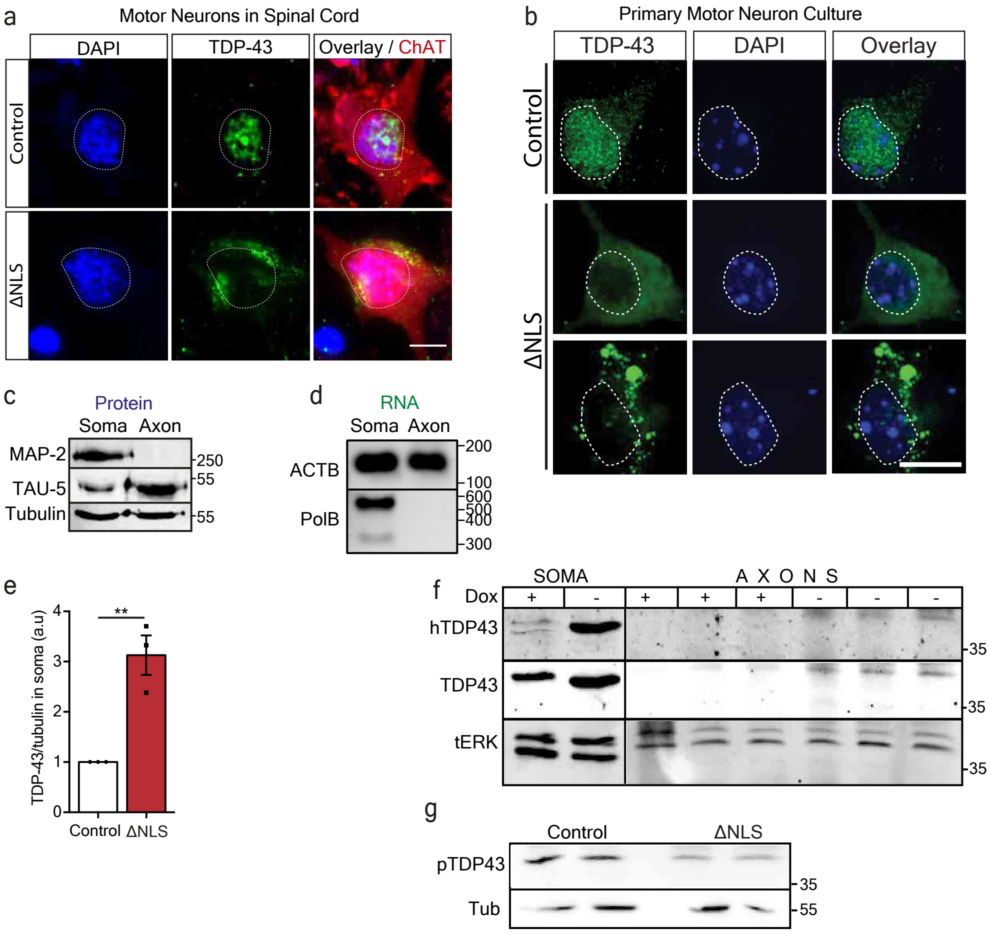

Supplementary figure 2 – TDP-43 mis-localizes in TDPΔNLS mice in-vivo and in-vitro.

a) Representative images of in-vivo SC MNs from TDPΔNLS-ChAT^{tdTomato} mouse and control stained with tTDP-43 antibody (green) and DAPI (blue). ChAT (red). Scale bar=10µm. n=3,3 mice. **b)** Representative images of TDPΔNLS primary cultured MNs cell bodies with (upper panel-control) or without dox (middle and lower panel), stained with TDP-43 antibody (green) and DAPI (blue). Scale bar=10µm. n=3 independent experiments. **c)** Western blot and **d)** PCR of axons extracted from radial MFCs. **In c**, MN axons and soma were blotted for MAP-2 (upper panel) to mark dendrites and TAU-5 (middle panel) to mark axons. Tubulin (lower panel) was used as a loading control. **In d**, Qualitative RT-PCR was performed for cell body-specific Polymerase B (Pol-B) as a control for fraction purity, together with Beta-Actin as a positive control. n=3 independent experiments. **e)** Western blot analysis of the normalized overall TDP-43 protein levels in control MNs or upon induction of TDPΔNLS expression. SE. n=3 repeats. Unpaired t-test, two-sided. **p=0.0056. **f)** Western blots showing all three repeats of hTDP43 and TDP43 levels in distal axons of control or TDPΔNLS MNs cultured in radial MFC. Total ERK (ERK1/2) was used for loading control. n=3 experiments. **g)** Full-uncropped blot of pTDP43 levels in distal axons of control or TDPΔNLS MNs cultured in radial MFC. Tubulin was used as loading control. n=3 experiments. a.u stands for arbitrary units. Source data are provided as a Source Data file.

Sup. Fig. 3

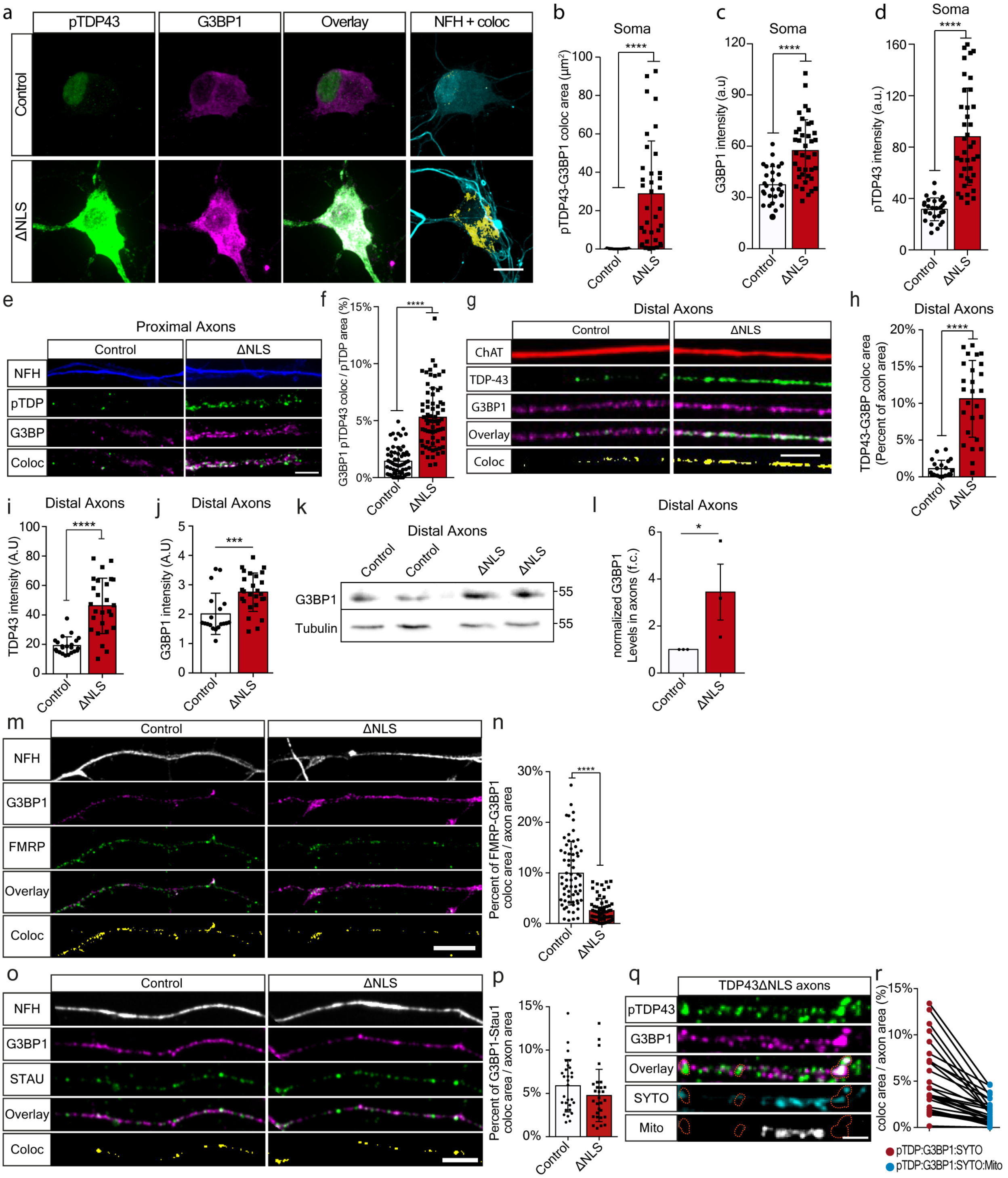

Supplementary figure 3 – TDP-43 cytoplasmic mislocalization leads to co-localization with RNP granule marker G3BP1 in Cell bodies, proximal and distal axons of TDPΔNLS MNs.

a) Representative images and **b)** quantification results from 3D-colocalization analysis examining the colocalization (yellow) of phosphorylated TDP-43 (pTDP; green) and G3BP1 (magenta) in cell bodies of cultured control or TDPΔNLS MNs. Cyan indicates NFH. Scale bar=10μm. n=38,40 cells. SD. Unpaired t-test, two-sided. ****p<0.0001. **c-d)** Quantification of G3BP1 (C) and pTDP43 (D) intensity in control or TDPΔNLS MNs. n=38,40 cells. Data is shown as the mean ± SD. Unpaired t-test, two-sided. ****p<0.0001. **e)** Representative images and **f)** quantification of the pTDP43-G3BP1 colocalization in

proximal axons of TDPΔNLS and control MNs. Blue indicates NFH, Green indicates pTDP43, Magenta indicates G3BP1. Scale bar=5μm. n=66,70 axons. SD. Unpaired t-test, two-sided. ****p<0.0001. **g-j**) Representative images (**g**) and quantification (**h-j**) of TDP-43 (green) and G3BP1 (magenta) colocalization (h), average TDP-43 (i) and average G3BP1 (j) intensities in distal axons of ChAT::Rosa (red) expressing TDPΔNLS or control MNs. Scale bar=10μm. n=22,26 axons. SD. Unpaired t-test, two-sided. ****p<0.0001, ***p=0.0008. **k)** Representative western-blot and **l)** quantification of G3BP1 levels in distal axons of control or TDPΔNLS cultured MNs within radial MFCs. Tubulin was used for loading control. n=3 experiments. SE. Unpaired Mann-Whitney test. *p=0.05. **m-n)** Representative images (m) and quantification (n) results from 3D-colocalization analysis examining the colocalization (yellow) of FMRP (green) and G3BP1 (magenta) in axons of cultured control or TDPΔNLS MNs. White indicates NFH. Scale bar=10μm. n=68,83 axons. SD. Unpaired t-test, two-sided. ****p<0.0001. **o-p)** Representative images (o) and quantification (p) results from 3D-colocalization analysis examining the colocalization (yellow) of Staufen1 (green) and G3BP1 (magenta) in axons of cultured control or TDPΔNLS MNs. White indicates NFH. Scale bar=5μm. n=29,29 axons. SD. Unpaired t-test, two-sided. **q-r)** Representative images (q) and colocalization analysis (r) showing pTDP (green), G3BP1 (magenta), Syto RNA (cyan) and mitotracker (white) staining in axons of cultured control or TDPΔNLS MNs. Scale bar=3μm. n=28,28 axons. a.u stands for arbitrary units. f.c stands for Fold Change. Source data are provided as a Source Data file.

Sup. Fig. 4

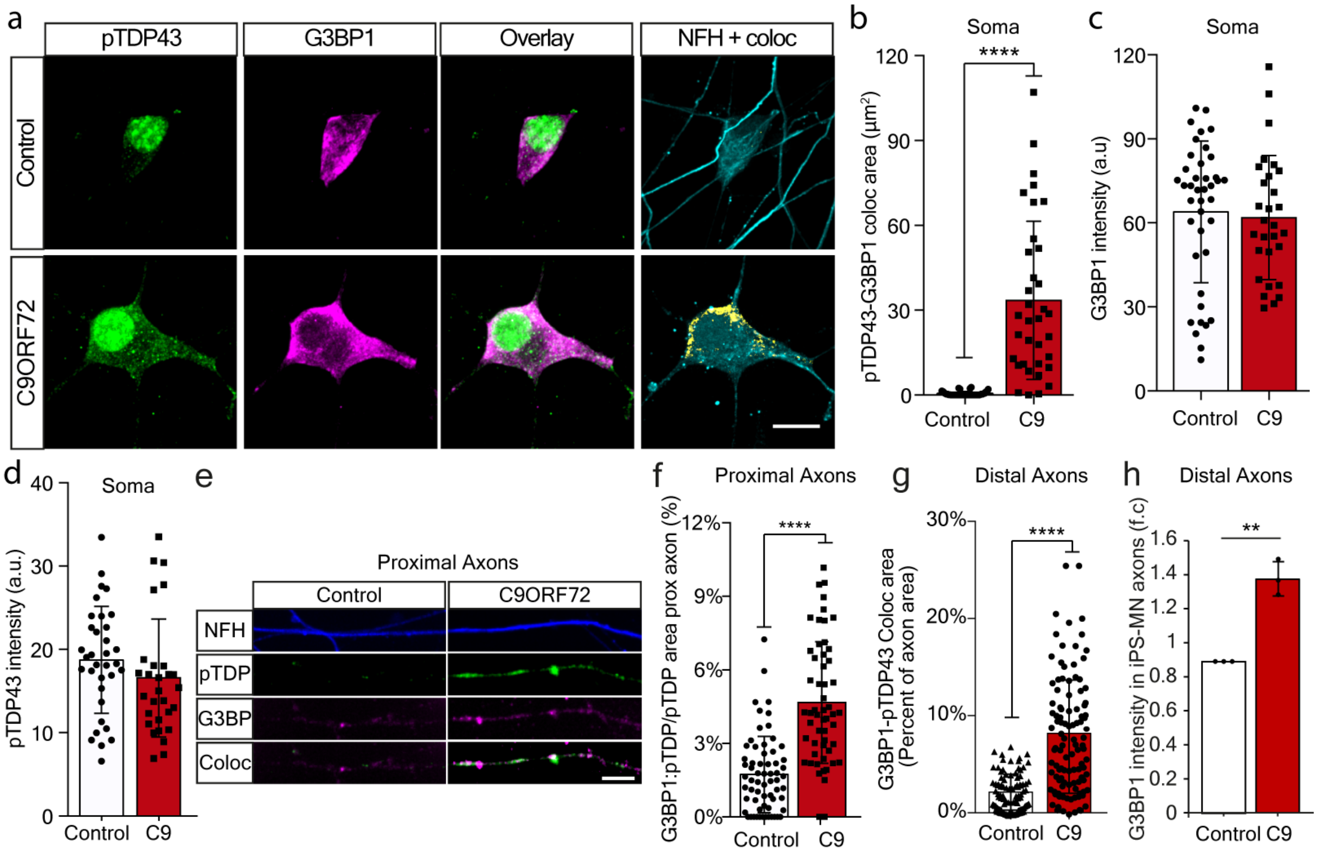

Supplementary figure 4 – Cytoplasmic mislocalization of phosphorylated-TDP-43 leads to co-localization with RNP granule marker G3BP1 in cell bodies, proximal and distal axons of ALS C9ORF72 iPS-MN.

a) Representative images and **b)** quantification results from 3D-colocalization analysis examining the colocalization (yellow) of phosphorylated TDP-43 (pTDP; green) and G3BP1 (magenta) in cell bodies of cultured C9ORF72 or isogenic control iPS-MNs. Cyan indicates NFH. Scale bar=10μm. n=46,35 cells. SD. Unpaired t-test, two-sided. ****p<0.0001. **c-d)** Quantification of G3BP1 (C) and pTDP43 (D) intensity in C9ORF72 or control iPS-MNs. n=46,35 cells. SD. **e)** Representative images and **f)** quantification of the pTDP43-G3BP1 colocalization in proximal axons of C9ORF72 and control MNs iPS-MNs. Blue indicates NFH, Green indicates pTDP43, Magenta indicates G3BP1. Scale bar=5μm. n=64,55 axons. SD. Unpaired t-test, two-sided. ****p<0.0001.

g-i) Quantification of G3BP1 colocalization and intensity in C9ORF72 and control iPS-MN **h**) The percent of TDP43-G3BP1 colocalized area out of the total axonal area in C9ORF72 and control iPS-MN. n=108,104 axons. SD. Unpaired t-test, two-sided. ****p<0.0001. **i)** Average G3BP1 intensity in iPS-MN axons n=4 repeats. SE. Unpaired t-test, two-sided. *p=0.028. a.u stands for arbitrary units. f.c stands for Fold Change. Source data are provided as a Source Data file.

Sup. Fig. 5

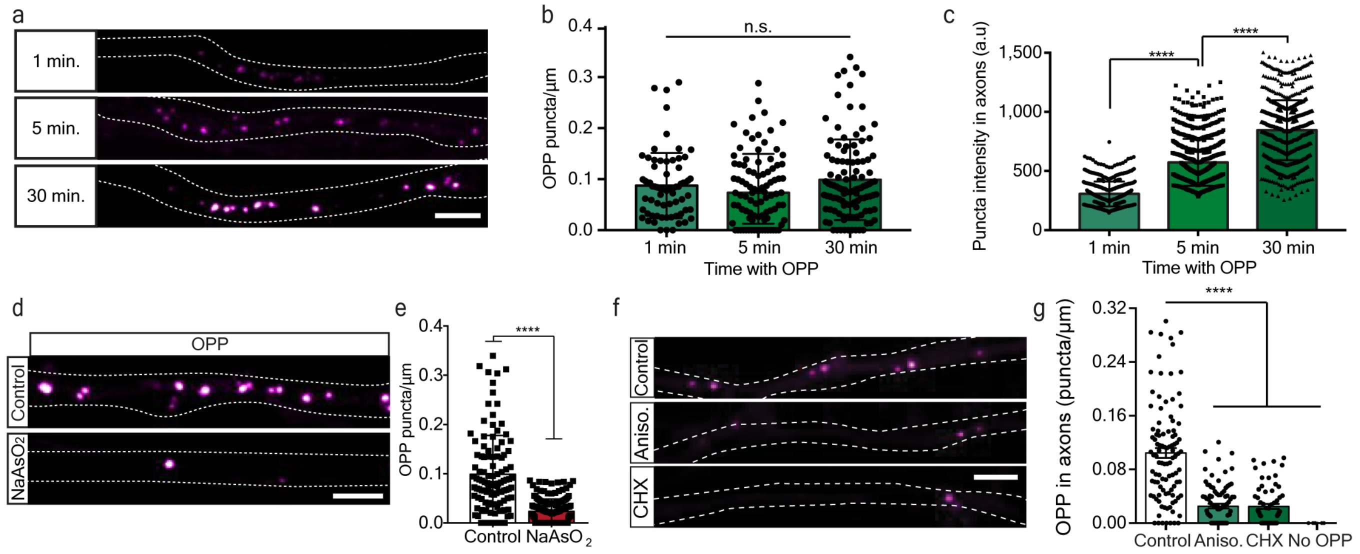

<u>Supplementary figure 5 – OPP Labeling in MFCs expose local protein synthesis in MN distal axons.</u>

a) Representative images and **b)** quantification of OPP density and **c)** OPP puncta fluorescence intensity in distal axons in MFC labeled with OPP (20µM) for different time periods (1 min, 5 min, 30 min). Scale bar=10µm. SD. b) n=70, 92, 110 axons. c) n=472, 741, 819 puncta. One-way ANOVA with Holm-Sidak correction. ****p<0.0001. **d**) Representative images and **e)** Quantification of OPP puncta in axons without (control) or with application of $NaAsO_2$ (250µM) to axonal compartment of MFC. Scale bar=10µm. n=110,137 axons. SD. One-way ANOVA with Holm-Sidak correction. ****p<0.0001. **f**) Representative images **g**) and quantification of OPP puncta in axons without (control) or with application of protein synthesis inhibitors Cycloheximide (CHX) and Anisomycin (Aniso). An additional control, with only color labeling but no puromycin (no OPP) was included. Scale bar=10µm. n=102,63,73,6 axons. One-way ANOVA with Holm-Sidak correction. ****p<0.0001. a.u stands for arbitrary units. Source data are provided as a Source Data file.

Sup. Fig. 6

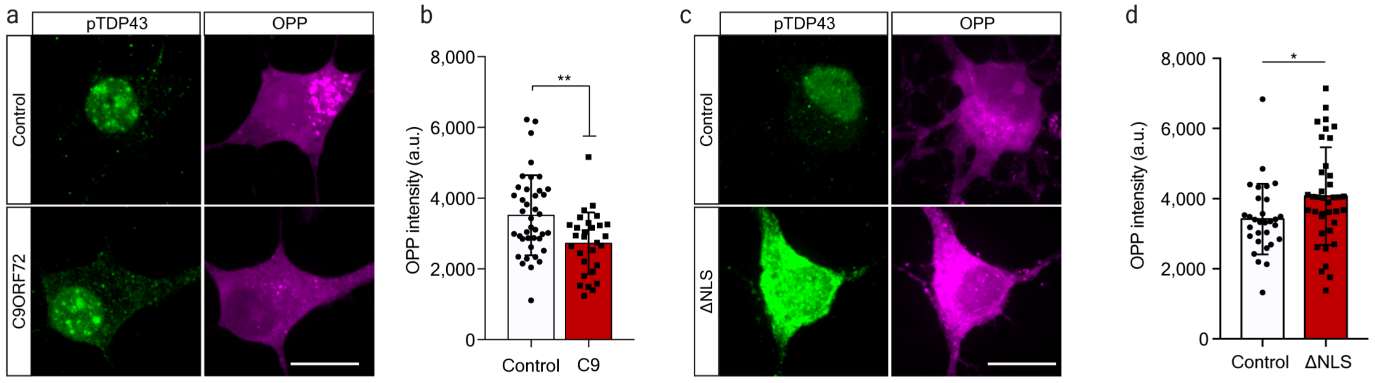

<u>Supplementary figure 6 – OPP Labeling of cell-bodies of C9ORF72 iPS-MNs and TDPΔNLS MNs.</u>

a) Representative images and **b)** quantification of OPP signal intensity in C9ORF72 and control iPS-MNs cell-bodies. Green indicates pTDP43, magenta indicates OPP. Scale bar=10μm. n=40,29. SD. Unpaired t-test, two-sided. **p=0.0025. **d)** Representative images and **c)** quantification of OPP signal intensity in TDPΔNLS and control MNs cell-bodies. Green indicates pTDP43, magenta indicates OPP. Scale bar=10μm. n=30,38 cells. SD. Unpaired t-test, two-sided. *p=0.034. a.u stands for arbitrary units. Source data are provided as a Source Data file.

Sup. Fig. 7

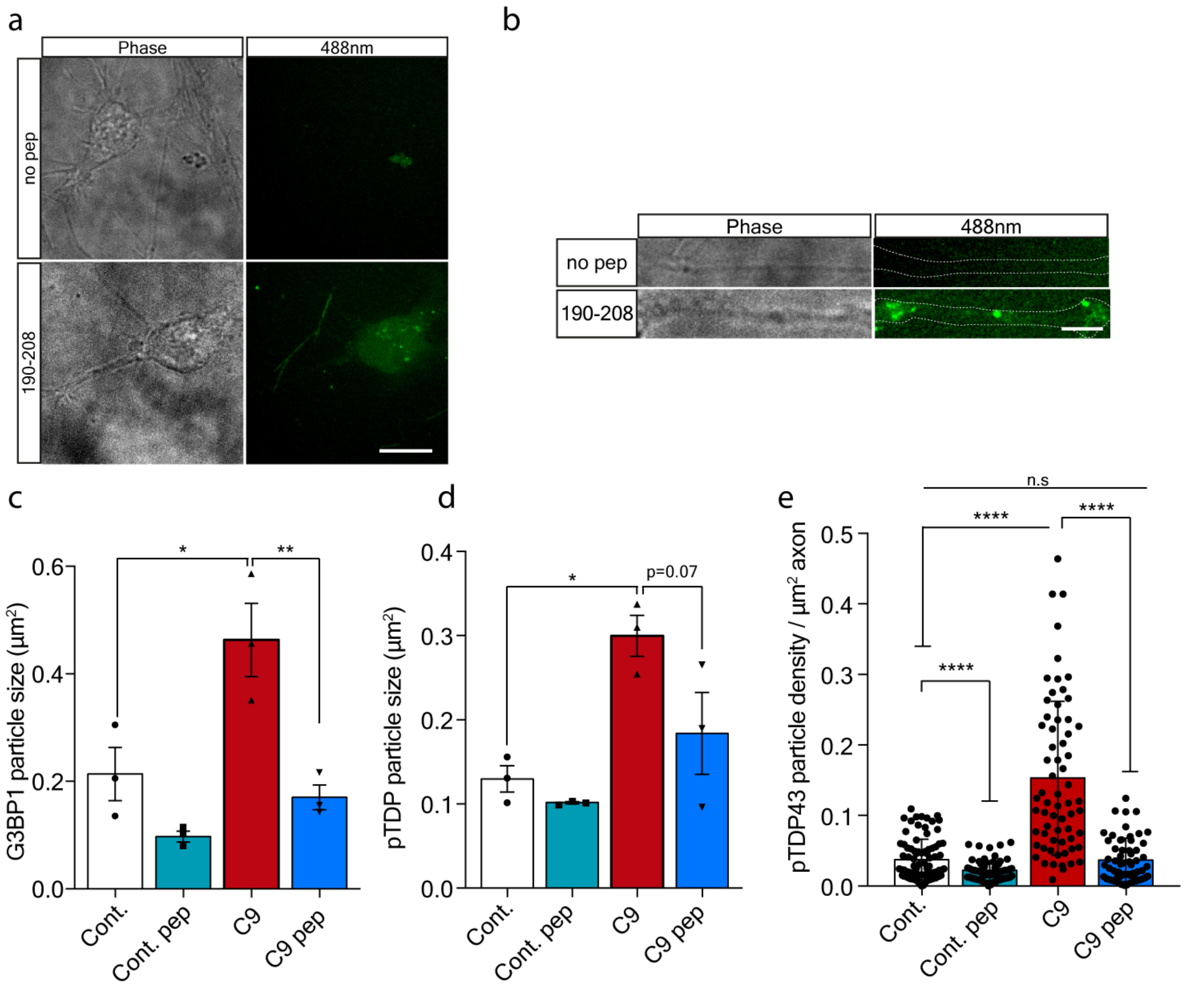

<u>Supplementary Figure 7 – G3BP1(190-208) Peptides Reduce G3BP1/pTDP43 particle size and pTDP43 RNP-condensate density in axons of C9ORF72 iPS-MNs.</u>

a-b) Representative phase and fluorescent images demonstrating penetration of TAT-fused G3BP1 (190-208)-FITC peptides into primary MNs cell bodies (a) and axons (b). Scale bar=10µm (a), 5µm (b). n=3 independent experiments. **c)** Quantification of G3BP1 particle size in C9ORF72 or control MN axons treated with G3BP1 peptides exclusively in the axonal compartment of the MFC. n=3 experiments, with more than 1,000 G3BP1 particles analyzed in each. SE. One-way ANOVA with Holm-Sidak correction. *p=0.0151, **p=0.0074. **d)** Quantification of pTDP-43 particle size in C9ORF72 or control MN axons treated with G3BP1 peptides exclusively in the axonal compartment of the MFC. n=3 experiments, with more than 1,000 pTDP-43 particles analyzed in each. SE. One-way ANOVA with Holm-Sidak correction. *p<0.0139. **e)** Quantification of pTDP-43 particle density in C9ORF72 or control MN axons treated with G3BP1 peptides exclusively in the axonal compartment of the MFC. n=91,71,65,68 axons. SD. One-way ANOVA with Holm-Sidak correction. ****p<0.0001. Source data are provided as a Source Data file.

Sup. Fig. 8

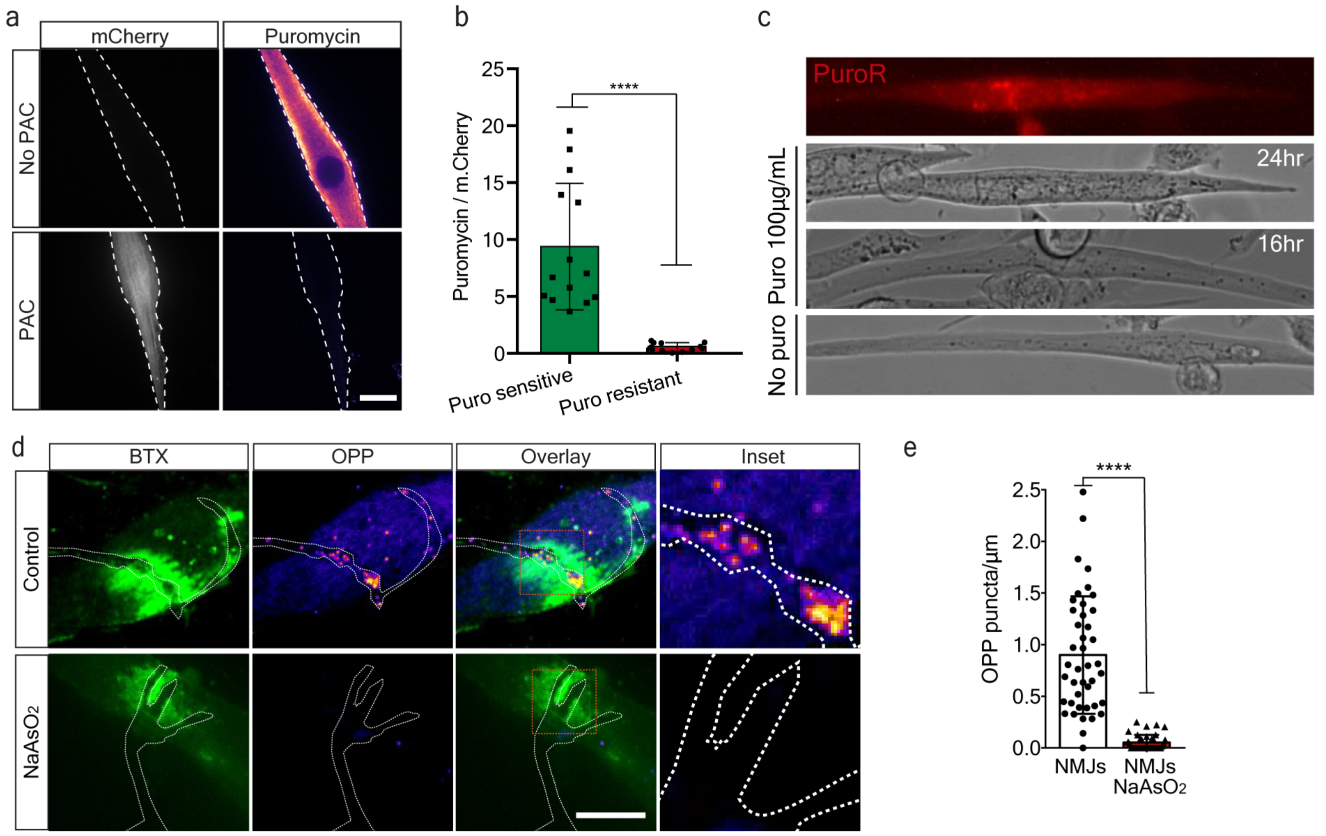

Supplementary Figure 8 – Puromycin-resistant muscles enable visualization of pre-synaptic protein synthesis in *in-vitro* NMJs.

a) Representative images and **b)** quantification of puromycin labeling in control, versus puromycin resistant muscles (transfected with PQCXIP-mCherry backbone vector expressing Puromycin Acetyltransferase gene (PAC)) demonstrating the ability of PAC to prevent puromycin labeling of newly synthesized peptides. Scale bar=20µm. n=14,14 muscles. Unpaired t-test, two-sided. ****$p<0.0001$. **c)** Representative images of puromycin-resistant muscles stable morphology following 16h and 24hr incubation with 100µg/mL puromycin compared to muscles that were not exposed to puromycin. **d)** Representative images and **e)** quantification of OPP puncta density of *in-vitro* NMJs in the presence of absence of distal $NaAsO_2$. Scale bar=5µm. n=42,44 NMJs. SD. Unpaired t-test, two-sided. ****$p<0.0001$. Source data are provided as a Source Data file.

Sup. Fig. 9

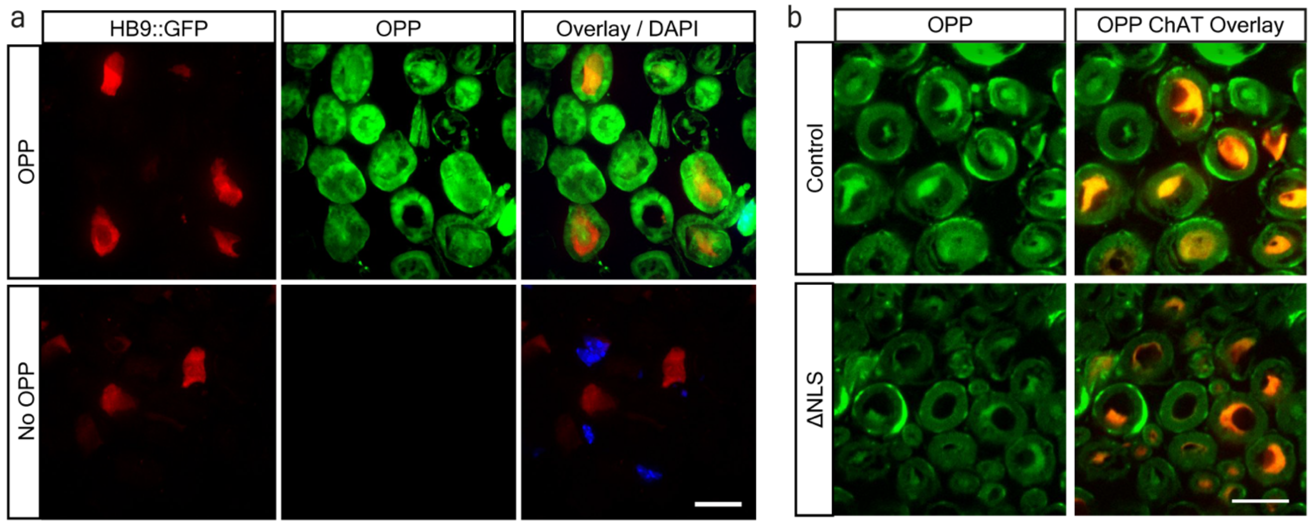

<u>Supplementary Figure 9 – OPP labeling in sciatic nerves reveals reduced axonal protein synthesis in TDPΔNLS MN axons.</u>

a) Representative images of OPP labeling versus only color labeling but no puromycin (no OPP) in HB9:GFP mouse SN sections. HB9 (red) indicates MN axons. Scale bar=10µm. n=3,3 mice. **b)** Additional unmasked OPP images and overlay images of SN sections obtained from TDPΔNLS and control mice. Scale bar=10µm. n=3,3 mice.

Sup. Fig. 10

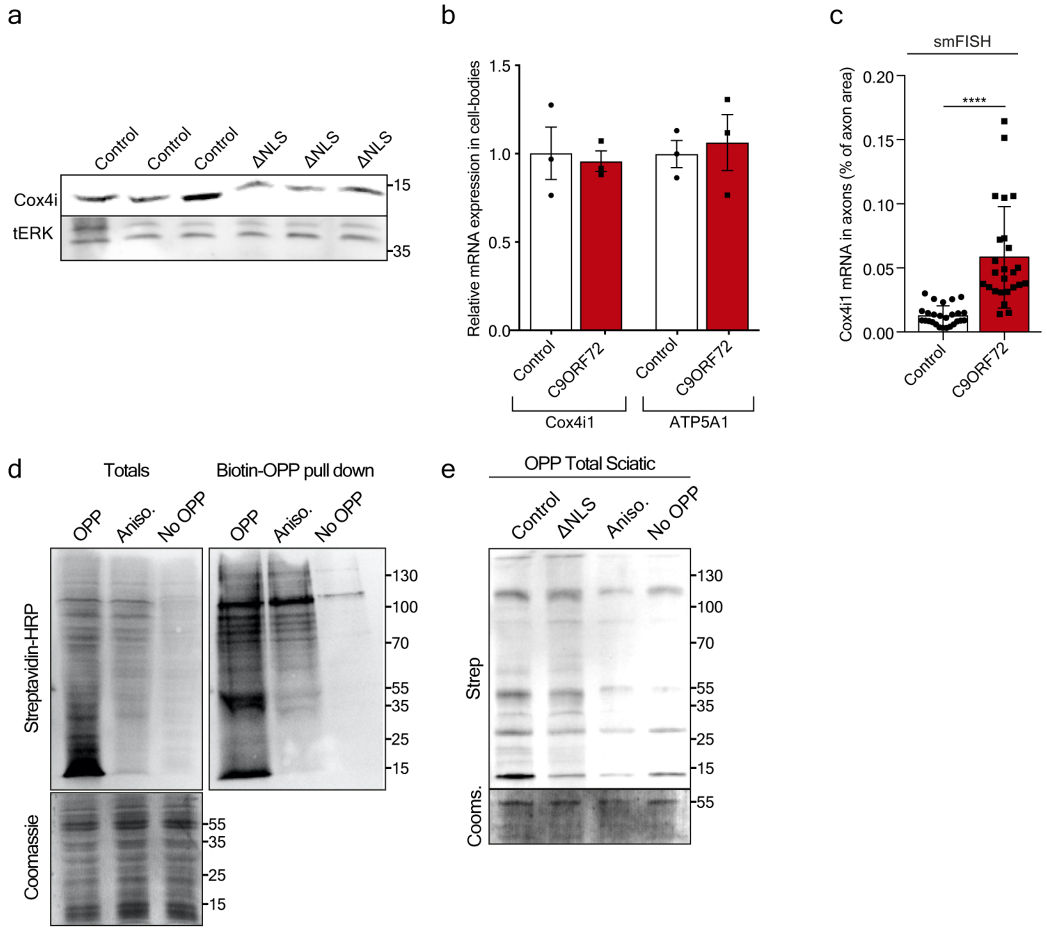

Supplementary Figure 10 - TDP-43 RNP-condensates limit the axonal synthesis of nuclear-encoded mitochondrial proteins.

a) Uncropped western blot of Cox4i protein levels in isolated axons of TDPΔNLS MNs cultured in radial MFC. n=3 experiments. **b)** RT-qPCR of Cox4i1 and ATP5A1 mRNA levels from somata of C9ORF72 and control iPS-MNs. n=3 independent repeats. SE. Unpaired-t-test, two-sided. **c)** quantification of Cox4i1 mRNA abundance in C9ORF72 and control iPS-MN axons. n=24,25 images from 3 repeats. SD. Unpaired-t-test, two-sided ****p<0.0001. **d)** OPP pull-down controls in HEK293T cells validation for specificity and sensitivity of assay. Left panel: Streptavidin-HRP (top) and Coomassie stained blots (bottom) of input lysates (Totals) from OPP-treated cultures compared to Anisomycin (Aniso.) and No-OPP controls. Right panel: Streptavidin-HRP blot of same samples following Biotin-OPP streptavidin pull-down. n=1 experiment per condition **e)** Streptavidin-HRP (top) and Coomassie stained (bottom) blots from OPP-labeled sciatic nerve axoplasms of Control, TDPΔNLS, Anisomycin-treated, and no-OPP controls. n=6 sciatic nerves (from 3 mice) per lane. Source data are provided as a Source Data file.

Sup. Fig. 11

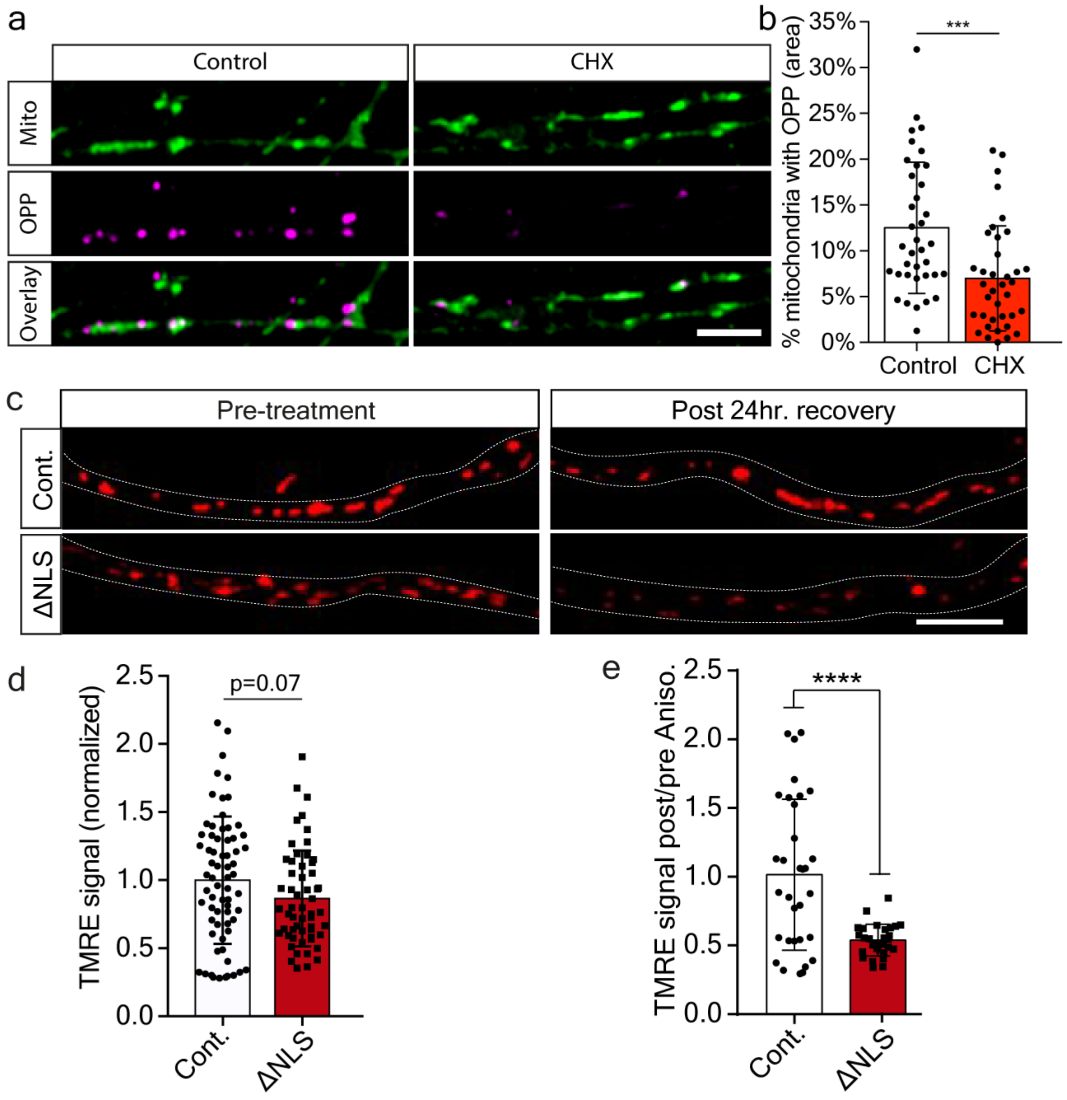

Supplementary Figure 11 - Mitochondria activity is dependent on local protein synthesis in MN axons, and is impaired in TDPΔNLS MNs

a) Representative images and **b**) quantification of the percent of mitochondria (area; green)) colocalization with OPP (area; red) in MN axons treated exclusively with cycloheximide (CHX) versus control (No CHX). Scale bar=5μm. n=38,38 axons. SD. Unpaired t-test, two-sided. ***p=0.0005. **c)** Representative images **d)** and quantification of the TMRE signal in TDPΔNLS and control MN axons. Scale bar=10μm. n= 68,56 axons. SD. Unpaired t-test, two-sided. p=0.0791. **e)** Quantification of TMRE signal in TDPΔNLS and control MN axons, following transient 4-hour anisomycin treatment and washout. TMRE signal was collected from distal axons 24-hours after washout and compared to pre-treatment signal. n=32,29 axons. SD. Unpaired-t-test, two-sided. ****p<0.0001. Source data are provided as a Source Data file.

Sup. Fig. 12

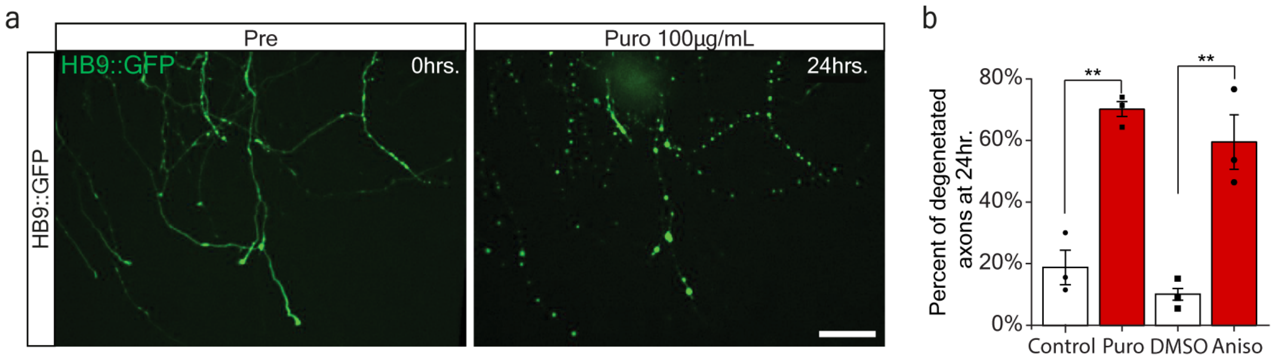

<u>Supplementary Figure 12 - local protein synthesis inhibition leads to axon degeneration.</u>

a) Representative images and **b**) quantification of the percent of degenerating HB9:GFP MN axons in the distal compartment of MFC following 24h axonal incubation with protein synthesis inhibitor Puromycin. DMSO application was used as a control for anisomycin treatment. Scale bar=100µm. n=3,3,3,3 experiments. SE. Unpaired t-test, two-sided. **p=0.067(left), **p=0.0013(right). Source data are provided as a Source Data file.

Sup. Fig. 13

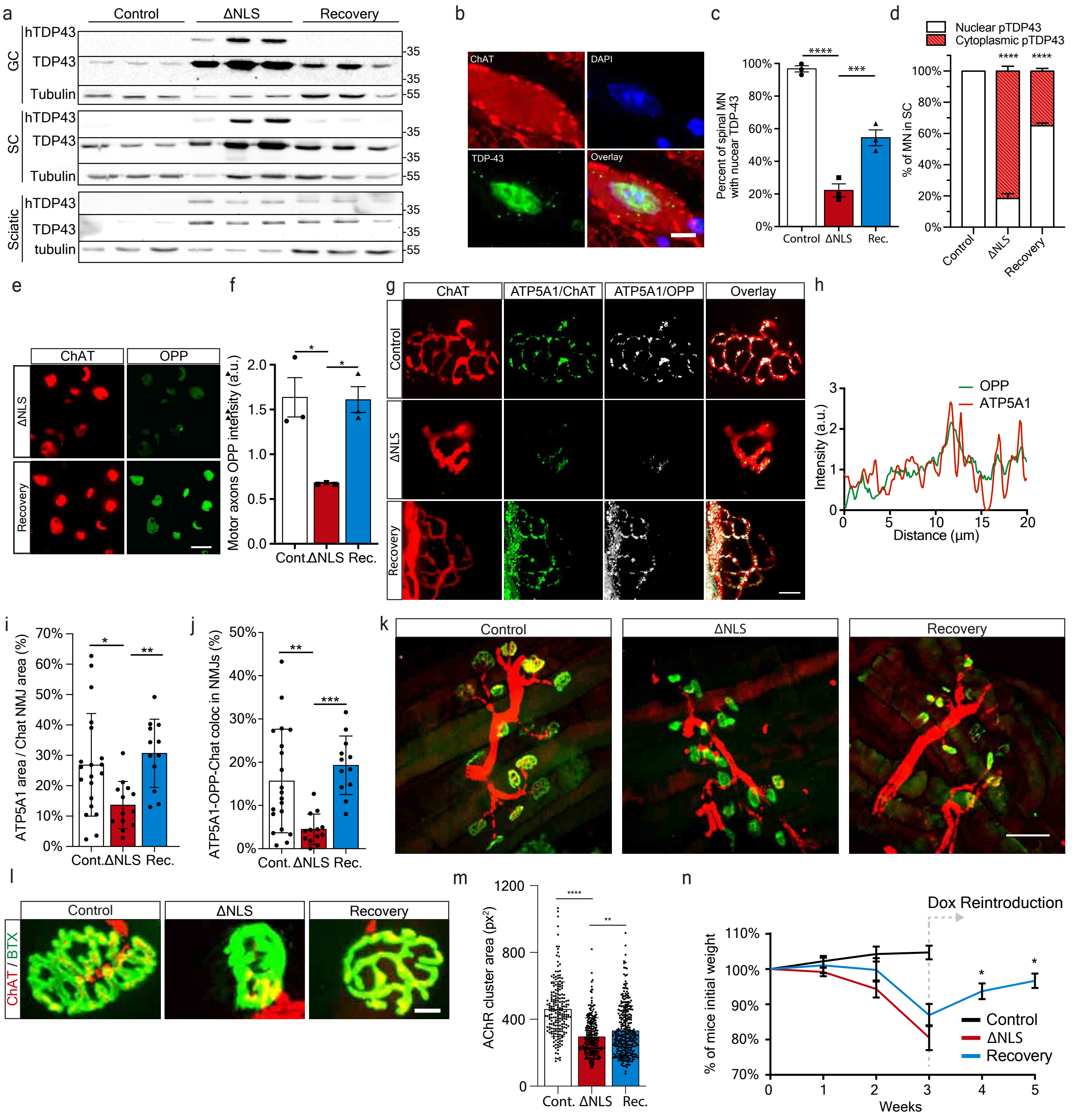

Supplementary Figure 13 – Dox re-application restores TDP-43 localization, reverses local synthesis inhibition and rescues TDP-43 mediated toxicity.

a) Western-blots for total-TDP-43 and human-TDP-43 (hTDP-43) in GC muscles, spinal cords (SC) and sciatic nerves of control, TDPΔNLS, and recovery mice. Tubulin was used as loading control. n=3 mice per condition (1 mouse per lane). **b)** Representative images and **c)** Quantitative analysis of the percent of spinal cord MNs (ChAT-red) with nuclear-localized TDP-43 in recovered mice compared to TDPΔNLS mice with no recovery. Nuclear localization was marked by colocalization of DAPI (blue) staining with TDP-43 (green). SE. Scale bar=5µm. n=3,3,3 mice. one-way ANOVA with Holm-Sidak correction. ***p=0.0010, ****p<0.0001. **d)** Quantitative analysis of the percent of MNs (ChAT-red) with nuclear versus cytoplasmic localization of pTDP-43 in spinal cords of control, TDPΔNLS, and recovery mice. n=3,3,3

mice. SE. One-way ANOVA with Holm-Sidak correction. ****p<0.0001. **e)** Representative images and **f)** quantification of OPP signal (green) within ChAT axons (red) in sciatic nerves from recovered mice compared to control and ∆NLS mice. Scale bar=10µm. SE. n=3,3,3 mice. One-way ANOVA with Holm-Sidak correction. *p=0.0166(left,right). **g)** Images and **h)** representative channel histograms of ATP5A1 (red) and OPP (green) intensities within pre-synaptic axon (ChAT) in NMJs of control TDP∆NLS mice, and **i-j)** colocalization analysis of the percent of ATP5A1 area (i) and ATP5A1 colocalization with OPP (j) within the pre-synapse area (ChAT) in TDP∆NLS mice compared with control and recovered mice. SD. n=20,13,12 NMJs. 3 mice from each group. One-way ANOVA with Holm-Sidak correction. ***p=0.0005, **p=0.0084(i), 0.0023(j), *p=0.0167. **k)** Representative images of NMJ innervation from TDP∆NLS mice compared with control and recovered mice. Scale bar=100µm. n=3 mice per condition. **l)** Representative images and **m)** quantitative analysis of BTX (green) post synaptic area cluster size in GC muscle NMJs in recovered mice as compared with ∆NLS and LM mice. ChAT signal (red) marks pre-synaptic innervation. Sacle bar=10µm. SD. n=202,263,318 NMJs. One-way ANOVA with Holm-Sidak correction. **p=0.0013, ****p<0.0001. **n)** TDP∆NLS, control and recovered mice weight measurements after dox retraction. Dox was introduced back to recovered mice at week 3. SE. n=18,15,9 mice for control, TDP∆NLS and recovery groups. One-way ANOVA with Holm-Sidak correction. *p=0.0194(left and right). a.u stands for arbitrary units. Source data are provided as a Source Data file.
