# Supplementary material for: Axonal TDP-43 condensates drive neuromuscular junction disruption through inhibition of local synthesis of nuclear encoded mitochondrial proteins
Source: Nat Commun. 2021 Nov 25;12:6914. doi: 10.1038/s41467-021-27221-8 (PMC8617040; doi:10.1038/s41467-021-27221-8)
Supplement: Supplementary file 2 — Reporting Summary [file 41467_2021_27221_MOESM2_ESM.pdf]

nature research

Corresponding author(s): Eran Perlson
Last updated by author(s): Oct 25, 2021

Reporting Summary

Nature Research wishes to improve the reproducibility of the work that we publish. This form provides structure for consistency and transparency in reporting. For further information on Nature Research policies, see our Editorial Policies and the Editorial Policy Checklist.

Statistics

For all statistical analyses, confirm that the following items are present in the figure legend, table legend, main text, or Methods section.

n/a	Confirmed	
☐	☒	The exact sample size (*n*) for each experimental group/condition, given as a discrete number and unit of measurement
☐	☒	A statement on whether measurements were taken from distinct samples or whether the same sample was measured repeatedly
☐	☒	The statistical test(s) used AND whether they are one- or two-sided<br>*Only common tests should be described solely by name; describe more complex techniques in the Methods section.*
☒	☐	A description of all covariates tested
☐	☒	A description of any assumptions or corrections, such as tests of normality and adjustment for multiple comparisons
☐	☒	A full description of the statistical parameters including central tendency (e.g. means) or other basic estimates (e.g. regression coefficient) AND variation (e.g. standard deviation) or associated estimates of uncertainty (e.g. confidence intervals)
☐	☒	For null hypothesis testing, the test statistic (e.g. F, t, r) with confidence intervals, effect sizes, degrees of freedom and P value noted<br>*Give P values as exact values whenever suitable.*
☒	☐	For Bayesian analysis, information on the choice of priors and Markov chain Monte Carlo settings
☒	☐	For hierarchical and complex designs, identification of the appropriate level for tests and full reporting of outcomes
☒	☐	Estimates of effect sizes (e.g. Cohen's d, Pearson's r), indicating how they were calculated

Our web collection on statistics for biologists contains articles on many of the points above.

Software and code

Policy information about availability of computer code

Data collection	Andor IQ V.3.0, FIJI V.2.0.0, Bitplane Imaris 8.4.3, MaxQuant V.1.5.3.8
Data analysis	Prism Graphpad V.8.0, Perseus V.1.6.2.3

For manuscripts utilizing custom algorithms or software that are central to the research but not yet described in published literature, software must be made available to editors and reviewers. We strongly encourage code deposition in a community repository (e.g. GitHub). See the Nature Research guidelines for submitting code & software for further information.

Data

Policy information about availability of data

All manuscripts must include a data availability statement. This statement should provide the following information, where applicable:
- Accession codes, unique identifiers, or web links for publicly available datasets
- A list of figures that have associated raw data
- A description of any restrictions on data availability

The proteomics data have been deposited to the ProteomeXchange Consortium via the PRIDE partner repository with the dataset identifiers PXD021876. Acquired proteomics data was correlated to the mouse FASTA databased using MaxQuant (v. 1.5.3.8) and its implemented Andromeda search engine.

Field-specific reporting

Please select the one below that is the best fit for your research. If you are not sure, read the appropriate sections before making your selection.

☒ Life sciences ☐ Behavioural & social sciences ☐ Ecological, evolutionary & environmental sciences

For a reference copy of the document with all sections, see nature.com/documents/nr-reporting-summary-flat.pdf

Life sciences study design

All studies must disclose on these points even when the disclosure is negative.

Sample size	We chose to use sample size of at least three independent repeats which is sufficient to determine statistical significance.<br>For in vivo experiment at least three mice from each group were measured.<br>In general, the number of animals in each group was determined according to previous studies cited in our manuscript.<br>For in vitro experiments at least 3 microfluidic chambers, each with at least 10 axons / NMJs from 3 independent biological repeats were used. High number of axons/NMJs was needed for immunoflourescent essays, to gain the full image of the axonal population, which is not homogeneous.<br>For muscle contraction essays, higher number of microfluidic chambers were used (7-10), as there is variability in this essay compared to other methods in this manuscript and as each chamber was counted as a whole rather than counting single axons.
Data exclusions	In rare cases, single outliers from technical repeats with extremely abnormal values due to technical issues were excluded automatically by outlier-identification, performed via Graphpad Prism based on standard deviation. This was done only when sample size was n>10. Exclusion was done via ROUT method; Q=1%.
Replication	To ensure reproducibility, all experiments were performed at least three independent times using similar conditions and reagents.<br>All attempts at replication were successful.
Randomization	For in vitro studies:<br>For TDP motor neurons, all cells were divided randomly to wells/chambers, half were treated with doxycyclin and half remained untreated to express the TDP mutation.<br>For HB9 motor neurons, cells were divided randomly between wells/chambers.<br>For IPSC derived motor neurons, cells were plated in similar numbers in wells/chambers based on known C9ORF72 or isogenic control cell genotype.<br>For in vivo studies:<br>Mice were predetermined by their genotype. For each experiment, to every randomly selected TDP mouse a littermate non-mutant control, age and sex matched, was assigned to serve as his biological control.<br>For human sample studies:<br>Samples from human participants were predetermined by their clinical diagnosis. The samples were labeled and slices from each patient were chosen randomly for the experimental immunoflourescent essay.
Blinding	Blinding was not applicable due to insufficient manpower. For every experiment, analysis was perfomed by the same person who obtained the data.

Reporting for specific materials, systems and methods

We require information from authors about some types of materials, experimental systems and methods used in many studies. Here, indicate whether each material, system or method listed is relevant to your study. If you are not sure if a list item applies to your research, read the appropriate section before selecting a response.

Materials & experimental systems

n/a	Involved in the study	
☐	☒	Antibodies
☐	☒	Eukaryotic cell lines
☒	☐	Palaeontology and archaeology
☐	☒	Animals and other organisms
☐	☒	Human research participants
☒	☐	Clinical data
☒	☐	Dual use research of concern

Methods

n/a	Involved in the study	
☒	☐	ChIP-seq
☒	☐	Flow cytometry
☒	☐	MRI-based neuroimaging

Antibodies

Antibodies used	Primary antibodies:<br>1. Phospho (409/410)-TDP43, Proteintech Cat. 22309-1-AP. For WB/IF - 1:2000<br>Validation for WB and IF - https://www.ptglab.com/products/phospho-409-410--TDP43-Antibody-22309-1-AP.htm#tested-

applications
2. TDP43, Proteintech. Cat. 10782-2-AP. For WB/IF - 1:2000. For pull down - 2μg per sample in 1ml.
Validation for WB, IP and IF - https://www.ptglab.com/products/TARDBP-Antibody-10782-2-AP.htm#tested-applications
3. TDP43 (Human specific), Proteintech. Cat. 60019-2-AP. For WB - 1:5000
Validation for WB - https://www.ptglab.com/products/TARDBP-Antibody-60019-2-Ig.htm#tested-applications
4. Neurofilament 200 (rabbit), Sigma. Cat. N4142. For IF - 1:500-1:1000
Validation for IF - https://www.sigmaaldrich.com/IL/en/product/sigma/n4142
5. Neurofilament 200 (Chicken), Abcam. Cat. ab72996. For IF 1:500-1:1000
Validation for IF based on 24 references appearing in - https://www.abcam.com/neurofilament-heavy-polypeptide-antibody-ab72996.html
6.Puromycin (clone 12D10), Millipore. Cat. MABE 343. For IF - 1:1000
Validation for IF - https://www.merckmillipore.com/INTL/en/product/Anti-Puromycin-Antibody-clone-12D10,MM_NF-MABE343?ReferrerURL=https%3A%2F%2Fwww.google.com%2F#anchor_Applications
7. ERK1/2 (065M4813V), Sigma. Cat. M5670. For WB - 1:10000
Validation for WB - 180 publications at https://www.sigmaaldrich.com/IL/en/product/sigma/m5670
8. alpha-Tubulin, Abcam. Cat. ab7291. For WB - 1:5000
Validation for WB - https://www.abcam.com/alpha-tubulin-antibody-dm1a-loading-control-ab7291.html
9. MAP2, Millipore. Cat. AB5622. For WB - 1:1000
Validation for WB - https://www.merckmillipore.com/INTL/en/product/Anti-MAP-2,MM_NF-AB5622-I?ReferrerURL=https%3A%2F%2Fwww.google.com%2F&bd=1#documentation
10. TAU-5, Abcam. Cat. ab80579. For WB - 1:250. For IF - 1:100
Validation for WB and IF - https://www.abcam.com/tau-antibody-tau-5-bsa-and-azide-free-ab80579.html
11. COX IV, Abcam. Cat. ab.16056. For WB - 1:300. For IF - 1:500
Validation for WB and IF - https://www.abcam.com/cox-iv-antibody-mitochondrial-loading-control-ab16056.html
12. ATP5A1 [EPR13030(b)], Abcam. Cat. ab176569. For IF - 1:500
Validation for IF - https://www.abcam.com/atp5a-antibody-epr13030b-ab176569.html
13. G3BP1 , Abcam. Cat. ab56574. For WB/IF - 1:1000
Validation for WB and IF - https://www.abcam.com/g3bp-antibody-2f3-ab56574.html

Secondary antibodies:
1. Goat anti chicken 405, Abcam. Cat. ab175675. For IF - 1:500
2. Goat anti chicken 488, Abcam. Cat. ab150173. For IF - 1:1000
3. Goat anti mouse 488. Abcam. Cat. ab150113. For IF - 1:1000
4. Goat anti mouse 594. Invitrogen. Cat. A11032. For IF - 1:1000
5. Goat anti mouse 650. Abcam. Cat. ab96882. For IF - 1:1000
6. Goat anti rabbit 488. Invitrogen. Cat. A11034. For IF - 1:1000
7. Goat anti rabbit 594. Jackson Laboratories. Cat. 111-585-144. For IF - 1:1000
8. Goat anti rabbit 640. Abcam. Cat. ab150083. For IF - 1:1000.
9. Donkey anti mouse HRP. Jackson Laboratories. Cat. 715-035-151. For WB - 1:10000.
10. Donkey anti rabbit HRP. Jackson Laboratories. Cat. 715-035-152. For WB - 1:10000.
11. Goat anti mouse IgG2a HRP. Jackson Laboratories. Cat. 115-035-206. For WB - 1:20000.

Validation

All antibodies were prevalidated by manufacturer, as stated in the supplied antibody data sheet and QA certificate.
Primary antibodies:
1. Phospho (409/410)-TDP43, Proteintech Cat. 22309-1-AP. WB/IF
Validation for WB and IF - https://www.ptglab.com/products/phospho-409-410--TDP43-Antibody-22309-1-AP.htm#tested-applications
2. TDP43, Proteintech. Cat. 10782-2-AP. WB/IF/pull down
Validation for WB, IP and IF - https://www.ptglab.com/products/TARDBP-Antibody-10782-2-AP.htm#tested-applications
3. TDP43 (Human specific), Proteintech. Cat. 60019-2-AP. WB
Validation for WB - https://www.ptglab.com/products/TARDBP-Antibody-60019-2-Ig.htm#tested-applications
4. Neurofilament 200 (rabbit), Sigma. Cat. N4142. IF
Validation for IF - https://www.sigmaaldrich.com/IL/en/product/sigma/n4142
5. Neurofilament 200 (Chicken), Abcam. Cat. ab72996. IF
Validation for IF based on 24 references appearing in - https://www.abcam.com/neurofilament-heavy-polypeptide-antibody-ab72996.html
6.Puromycin (clone 12D10), Millipore. Cat. MABE 343. IF
Validation for IF - https://www.merckmillipore.com/INTL/en/product/Anti-Puromycin-Antibody-clone-12D10,MM_NF-MABE343?ReferrerURL=https%3A%2F%2Fwww.google.com%2F#anchor_Applications
7. ERK1/2 (065M4813V), Sigma. Cat. M5670. WB
Validation for WB - 180 publications at https://www.sigmaaldrich.com/IL/en/product/sigma/m5670
8. alpha-Tubulin, Abcam. Cat. ab7291. WB
Validation for WB - https://www.abcam.com/alpha-tubulin-antibody-dm1a-loading-control-ab7291.html
9. MAP2, Millipore. Cat. AB5622. WB
Validation for WB - https://www.merckmillipore.com/INTL/en/product/Anti-MAP-2,MM_NF-AB5622-I?ReferrerURL=https%3A%2F%2Fwww.google.com%2F&bd=1#documentation
10. TAU-5, Abcam. Cat. ab80579. WB/IF
Validation for WB and IF - https://www.abcam.com/tau-antibody-tau-5-bsa-and-azide-free-ab80579.html
11. COX IV, Abcam. Cat. ab.16056. WB/IF
Validation for WB and IF - https://www.abcam.com/cox-iv-antibody-mitochondrial-loading-control-ab16056.html
12. ATP5A1 [EPR13030(b)], Abcam. Cat. ab176569. IF
Validation for IF - https://www.abcam.com/atp5a-antibody-epr13030b-ab176569.html
13. G3BP1 , Abcam. Cat. ab56574. WB/IF
Validation for WB and IF - https://www.abcam.com/g3bp-antibody-2f3-ab56574.html

Eukaryotic cell lines

Policy information about cell lines

Cell line source(s)	HEK293-T
Authentication	293-T cells were purchased from ATCC.
Mycoplasma contamination	Cell lines were routinly tested for mycoplasma, and found to be negative. We used mycoplasma detection kit (Biological Industries, Israel).
Commonly misidentified lines (See ICLAC register)	No misidentified cell lines were used.

Animals and other organisms

Policy information about studies involving animals; ARRIVE guidelines recommended for reporting animal research

Laboratory animals

1. NEFH-tTA line 8 (Jax Stock No: 025397) and B6;C3-Tg(tetO-TARDBP*)4Vle/J (Jax Stock No: 014650) were cross-bred to create NEFH-hTDP-43ΔNLS (TDP43ΔNLS) mice line.

2. ChAT::cre - tdTomato::lox- hTDP-43ΔNLS was obtained by crossing the hTDP-43ΔNLS with ChAT::cre and tdTomato::lox mice, (Jax stock no. 006410 and 007908, respectively).

3. HB9-GFP (Jax stock no. 005029)

4. Thy1-COX8A/Dendra (Jax stock no. 025401)

5. SOD1G93A (Jax stock No. 002726) mice were originally obtained from Jackson Laboratories and maintained by breeding with C57BL/6J mice. Only non-transgenic, p60 (C57BL/6J WT) females from this colony were used for the purpose of primary myocyte culture.

For in-vivo studies, mice were between the ages of 60-180days, mixed sex.
Littermate mice that are age and sex matched to mutant animals were used as controls.
Genotype was pre-validated via PCR.

The animals were grown at Tel Aviv SPF facility under strict hygiene standards. In the facility, Groups of up to five mice per cage were housed on a 12 h light/dark cycle, on autoclaved ASPEN wood chips bedding, at an ambient temperature of 22°C ±1°C, with humidity controlled at 50%, and were provided with UV-irradiated and micro-filtered Hydropac system for water.

Wild animals	This study did not involve wild animals
Field-collected samples	This study did not involve samples collected from the field.
Ethics oversight	All animal experiments were approved and supervised by the Animal Ethics Committee of Tel-Aviv University.

Note that full information on the approval of the study protocol must also be provided in the manuscript.

Human research participants

Policy information about studies involving human research participants

Population characteristics

muscle biopsies from 3 ALS patients (Males, ages 54, 75. Female, age 33) and 5 non-ALS patients (Male, age 66. Females, Ages 25, 25, 51, 81) were used. All patient were adults (18-81).
All 3 ALS patients were diagnosed with clinically definite or probable ALS according to El Escorial criteria.
Control muscles included a variation of findings, which were consistent with a diagnosis of normal muscle, severe, chronic ongoing denervation and reinnervation due to spinal stenosis, necrotic autoimmune myopathy, type 2 fiber atrophy due to disuse and overlap myositis syndrome.

Recruitment

All clinical and muscle biopsy materials used in this study were obtained with written informed consent during 2016-2020 for diagnostic purposes followed by research application.
There was no self selection bias. All available ALS samples were taken for the study, and control samples were chosen randomly from the sample bank that was approved for this current study, without any identifying features. Therefore, there should not be any effect for pre-selection bias on the results of this study.

Ethics oversight

Sheba medical center Helsinki institutional review board, Ramat-Gan, Israel.

Note that full information on the approval of the study protocol must also be provided in the manuscript.
