# Supplementary material for: Axonal TDP-43 condensates drive neuromuscular junction disruption through inhibition of local synthesis of nuclear encoded mitochondrial proteins
Source: Nat Commun. 2021 Nov 25;12:6914. doi: 10.1038/s41467-021-27221-8 (PMC8617040; doi:10.1038/s41467-021-27221-8)
Supplement: Supplementary file 4 — Description of Additional Supplementary Files [file 41467_2021_27221_MOESM4_ESM.pdf]

Title: Supplementary Movie 1 –
Description: OPP labeling in muscles identifies local protein synthesis in the pre-synaptic MN axon at the NMJ: 3D reconstitution of NMJ from hind-muscle demonstrating labeling of newly synthesized proteins in the pre-synaptic axon. ChAT is indicated by red color. OPP is indicated by green color. Colocalization of ChAT and OPP is indicated by yellow color.

Title: Supplementary Movie 2 –
Description: Pre irradiating pre-synaptic mitochondria inhibits NMJ function: Pre-synaptic MNs were infected with lentivirus encoding mito-killer-red (MKR) protein, and irradiated specifically at the muscle contact sites, the NMJs. Muscle calcium transients, indicating muscle activity, were imaged prior to MKR irradiation. 500-frame movies were acquired at 60 millisecond intervals for a total of 30 seconds.

Title: Supplementary Movie 3 –
Description: Post irradiating pre-synaptic mitochondria inhibits NMJ function: Pre-synaptic MNs were infected with lentivirus encoding mito-killer-red (MKR) protein, and irradiated specifically at the muscle contact sites, the NMJs. Muscle calcium transients, indicating muscle activity, were imaged 30 minutes after (post-Sup. Movie 3) MKR irradiation. 500-frame movies were acquired at 60 millisecond intervals for a total of 30 seconds.

Title: Supplementary Movie 4 -
Description: Normal NMJ activity in control neuromuscular cultures: Neuromuscular cocultures at 10 DIC labeled with OGB for visualizing axonal and muscular calcium transients. Arrowheads indicate presynaptic axon and muscle simultaneously become active, demonstrating undisrupted neuromuscular transmission. Scale bar=20μm.

Title: Supplementary Movie 5 -
Description: Dysfunctional NMJ activity in puromycin treated neuromuscular cultures: Neuromuscular co-cultures at 10 DIC labeled with OGB, 16 hours after puromycin (100μg/mL) was added exclusively to the distal/NMJ compartment of MFC. Arrowheads indicated calcium transient in presynaptic axon and unresponsive (inactive) muscle. Scale bar=20μm.

Title: Supplementary Movie 6 –
Description: Cox4i Enrichment in pre-synaptic axon at NMJs: 3D reconstitution of NMJ from EDL muscle demonstrates Cox4i enrichment in the presynaptic axon at the NMJ. ChAT is indicated by red color. Cox4i is indicated by green color. Colocalization of ChAT and Cox4i is indicated by yellow color.

Title: Supplementary Data 1 –
Description: Proteome analysis on sciatic axoplasm isolated from control and TDPΔNLS mice. Proteome analysis results including the list of proteins identified in sciatic axoplasm samples and their relative abundance in TDPΔNLS versus control mice. The list of significantly altered proteins was further aligned with Mitocarta 2.0 database to identify deregulation in nuclear encoded mitochondrial genes. n=4,4 mice.
